# Round the Hospitals

**Published:** 1919-02-15

**Authors:** 


### ROUND THE HOSPITALS.

It will be good news to the nurses of St. Bartholomew's Hospital, and indeed to all hospital workers who are interested in The Oldest Hospital Foundation we possess in the Metropolis, to learn that there are now excellent prospects for the New Nursing Home which has been urgently required at this institution, and has been declared by its Governors for upwards of forty years to be a first and most urgent need. Now, thanks to Lord Sandhurst, a movement is really being made which should speedily result in the provision of the £150,000 required. We venture to express a hope that the Governors of this institution, who comprise a considerable number of very wealthy men and women, should make it their business to provide the money in record time. The friends of St. Bartholomew's Hospital owe it to their reputation to make the gift worthy of their great institution

**ROUND THE HOSPITALS**—(*continued*).

and of the supreme claims which their nurses have upon the governors and the funds of this endowed hospital.

The delays which have taken place in the past have not added to the dignity, authority, or glory of St. Bartholomew's Hospital, and it would be a fitting celebration of the conclusion of peace to make the Nurses' Home the Governors' Memorial of the successful termination of the war and of the splendid services which have been rendered by British nurses. Nurses have given themselves to the cause of the nation and the succour, often in circumstances of great personal danger, of the brave men who, in God's good providence, have saved this nation and Empire from disasters and horrors which cannot be exaggerated. It is a great point that the actual amount required is known to be £150,000—a sum which should be well within the compass of those who will surely regard it as their privilege to subscribe without a moment's avoidable delay. Meanwhile we thank Lord Sandhurst for the step he has taken. We have confidence that he will have behind him the whole body of governors, so that the speedy erection and equipment of this great building when finished, may prove to be the Finest Nurses' Palace the world contains.

It is reassuring to learn that Miss Cummins, Lady Almoner of St. Thomas's Hospital, has been elected to serve on the Child-Welfare Committee of North Lambeth Borough Council. The appointment of women on these committees is provided for in the Maternity and Child-Welfare Act (1918). But it is not only women who are needed; it is a special type of woman fitted to place at the service of the committee advice founded on practical knowledge. St. Thomas's has long taken the lead among the Metropolitan hospitals in training women through their admirable Almoner's department for infant and maternal work, and Miss Cummins has won a high reputation by her development of this valuable department.

Nurses must have been greatly moved recently by the spectacle of men striking for the inclusion of mealtimes in an eight-hour day. Some, no doubt, of the women who suffered in the slush and fog for want of means of transport, had come out from a twelve hours' spell of night duty, unrelieved by even one hour out of the wards. Every victory of labour in the struggle for shorter hours and better conditions accentuates the indefensible length of the nurses' day, and still more of her night on duty. For nearly a century after the principle of State intervention in labour conditions had been recognised it was confined to the employment of women and children. We have now the amazing spectacle of a community which accedes to the demands of the men for lighter work, for seven days' work in six (or is it now five?) days a week, yet contentedly acquiescing the fact that by far the most popular and indispensable class of women workers are compelled to work something like ten hours a day for seven days in the week. The great thing to do is to keep this fact well before the public. Its driving force is irresistible.

The Fever Nurses' Association has promulgated a standardised rate of pay for fever nurses which they are asking all local authorities to adopt. It is on the principle now pretty generally established, that the larger the institution the higher the responsibilities of the matron and the higher the value of the work. For matrons of hospitals containing not less than 100 beds the scale provides £100 per annum. Every additional 100 beds adds an extra £25 to the stipend, until the maximum is reached at £200 in a hospital with 500 beds. Long service pay adds £2 to the salary after five years' service, and £4 after ten years' service. Sisters with general training and fever certificates are to have £46, rising by yearly increment of £2 to £50, a rate which does not strike us as munificent. Staff nurses are to begin at £32, and rise by yearly increment of £2 to £40. Probationers and junior nurses are to receive £20, £24. A war bonus is to be paid in addition to the above. It appears that the local authorities have accepted the scale as satisfactory. The Health Committee at East Ham suggests, however, increasing the present weekly war bonus to 6s.

The Mount Vernon Hospital at Northwood has made further important changes in salaries. The sisters' posts have now been made really good, rising as they do to £75. This is an exceedingly far sighted and wise measure since the whole tone of hospitals which keep their cases for a long time depends on getting and retaining at the head of the wards experienced women possessed of hearts and understanding. Probationers are to receive £20, £25, and £30.

Though we hear complaints from many quarters that advertisements for nurses produce few, if any, applications, the same thing does not hold good in regard to vacancies for health visitors. These posts appear to be in considerable demand, and to attract nurses engaged in every branch of the profession. Advertisements recently inserted by the Cardiff Corporation's Maternity Committee, for example, brought in a good crop of candidates, among the applicants being hospital sisters and staff nurses, private, maternity, and district nurses, inspectors of midwives and practising midwives, tuberculosis nurses, and so forth. The response may have been due in some measure to the salary attaching to the posts—£120 per annum, rising to £150—being more adequate than in some instances. But there is little doubt that health visiting is coming to be regarded by nurses as a congenial and very desirable sphere of work. Health visiting, too, seems to offer an opportunity for nurses who are married or who have been left widows. It is a [illegible]ther remarkable feature that more than one-third of those from whom the Cardiff selection was made were either married or widows.

ROUND THE HOSPITALS—(*continued*).

The St. Pancras Guardians have assumed an entirely unjustifiable attitude in regard to the exertions of their nursing staff in the recent epidemic and consequent period of overstrain. The Infirmary Committee presented, as it was their duty to do, a recommendation that gratuities should be granted to the Matron and her staff of nurses in recognition of their self-sacrificing exertions in the service of the sick poor when the infirmary was overcrowded, and many nurses and other workers were ill and off duty. Several members supported the motion, but it did not meet with approbation from the main body of Guardians present, and was referred back for further consideration. Now we learn that the Guardians have resolved on a truly munificent token of gratitude. Each nurse is to get—a letter of thanks! It is well the sands are running out and that the St. Pancras worthies will not long be entitled to interfere in the nursing affairs of this fine hospital. The staff will smile over their stereotyped letters. They will not be wasted, for they will serve to bring the claims of nurses to an eight-hour day and overtime pay very glaringly indeed before the public.

After a long and animated discussion the Wandsworth Guardians reversed the decision of their Committee to accept the resignation of Sister Elizabeth Molony, who for sixteen years has been in their service at the St. John's Hill Infirmary, during twelve years of which time she has been Charge-Nurse. Sister Molony had appealed for justice to the Board in a letter, in which she declared her resignation had been forced upon her by the Chairman, Canon Curtis. She stated that the feeding and other causes of dissatisfaction which had been complained of by one of the patients had been for months past periodically brought to the notice of Dr. Nixey by herself, but had been ignored. The evidence showed that she made a hasty remark to the doctor in support of a patient's complaints, and that she was subsequently recommended by Canon Curtis to resign on the ground that if the matter were brought before the Board she would be exposed to publicity. There seems to be no doubt she was led to believe that she must choose between resignation or dismissal. Mrs. Cassidy, with a refreshing sense of fair play, declared that an apology to the doctor would have settled the whole matter, and that it would be a blot on their escutcheon of fair administration if they let Sister Molony go after sixteen years' hard work without a single complaint being made against her. "I don't wonder they want to abolish us," she concluded. The Chairman appears to have lost his temper, and the contrast between the angry utterances of the ecclesiastic and the mild retort which Sister Molony permitted herself in addressing the doctor is sufficiently instructive.

We record with pleasure the spread of the one day in seven movement in South Wales. At a meeting of the House Committee of the Swansea General and Eye Hospital held on February 5 it was decided to grant the nursing staff one day off in seven so soon as the necessary increase in the number of probationers can be obtained. The training is for three years, and probationers receive £12, £15, £24. Nurses remaining for an extra (optional) year after certification receive £35. The ward and theatre sisters receive £50 to £60; the night superintendent and housekeeping sister £55 to £65. We feel confident that the institution will speedily feel the benefit of the managers' large-minded policy, which is calculated to attract a good class of probationer, and maintain a spirit of willing and vigorous energy among all members of the staff.

A great incubus has been taken off the Poor-Law institutions of the country by the circular letter addressed by the Local Government Board to boards of guardians authorising the managers to revert to the use of the dietary tables in force before the introduction of rationing. In butcher's meat, sugar, fats, and jam the present restrictions are still to be observed, nor can allowances of milk and cheese be increased. Rations of potatoes and vegetables as well as of bread may be issued "according to appetite," a highly sensible and economical arrangement, and by a rare stretch of imagination the Board revokes or rather "waives" the existing direction which permits a new or revised diet-table to come into operation only on quarter day. It has actually been discovered at headquarters that the character of the food supplies obtainable changes oftener than once in three months.

The King has been pleased to award the Royal Red Cross to the following ladies of the Nursing Services in recognition of their valuable services with the British Forces in East Africa:—First Class: Miss M. E. Ensor, matron, and Miss M. Gilkes Robinson, of the T.F.N.S.; Miss L. Outhwaite, matron, Q.A.I.M.N.S.I. Second Class: Miss E. B. Beet, staff nurse, and Miss M. McLachlan, sister, Q.A.I.M.N.S.R.; Mrs. N. Browne, V.A.D.; Mrs. K. Chisholm, matron, Northern Rhodesia Med. Co.; Miss M. Deiringer, sister; Miss K. L. Fletcher, sister, Miss J. Lambert, sister; Miss L. Lezard, staff nurse; Miss Q. May, sister; Miss E. B. Topp, sister; Miss C. Ward, staff nurse, all of the S.A.M.N.S.; Miss B. D. Lovibond, nursing sister, E. Afr. Mil. N.S.; Miss E. M. Pratt, sister, Uganda Med. Ser. Co.

Both in Durham and in Birmingham fine tributes have been paid in the course of the week to the voluntary auxiliary hospital work carried out by nurses and V.A.D. members during the war. Lord Durham presenting awards on February 1 to numerous V.A.D. members and others brought under review the work of the last four years, in which twenty-eight hospitals had been opened in Durham, 1,517 beds equipped, 37,704 patients treated, and 1,202 V.A.D. members passed to hospital service—a splendid record for the county. At

**ROUND THE HOSPITALS—(*continued*).**

Birmingham the workers associated with the Voluntary Aid Detachments were received by the Lord Mayor and Lady Mayoress at the Town Hall, on February 6, to receive the thanks of the city for the manifold services they have rendered to the wounded. The auxiliary hospitals in the city, fourteen or fifteen in number, have treated 22,169 patients. The Lord Mayor referred in warm terms to the services of the nurses who had trained and directed the V.A.D. members.

Severe strictures on two of the nursing staff of the Oswestry Cottage Hospital were passed by the local Coroner, who is a medical man, at an inquest which he held on February 4 on a boy of seven who died in the institution as a result of scalds. It appeared that the nurses—one of whom is acting-matron of the hospital—attended the child first at his home, and did not consult a medical man for a couple of days. The doctor ordered the boy's removal to the hospital, where, however, he died. Both nurses admitted that they were unqualified, and the Coroner told them they were not capable of taking charge of the case, and had assumed an unwarrantable responsibility. But what are the managers of the Oswestry Hospital about to staff the institution with untrained women? We hope an inquiry will be held by the subscribers into the circumstances.

A decision of some importance to nursing institutions was given recently in the Chancery Division by Mr. Justice Astbury. The issue raised was whether the Worcester City and County Nursing Institution was legally a charitable institution, and thereby entitled to retain as a charitable devise three acres of land and a house in Worcester left to it in trust by the late Amy Mary Wheeley. Mr. Justice Astbury held that it was a legal charitable institution, observing that it was clear the benefits the institution gave patients were given for fees considerably lower than those charged by ordinary nursing homes, and that no profits could be made by the owners.

At the annual prize-giving of the Tynemouth Poor-Law Hospital the Chairman spoke appreciatively of the success which had attended the formation of the training-school for nurses in the institution in 1911. The staff, which was then nineteen, has now increased to thirty, including war probationers. Preparation is given for the C.M.B. examinations, instruction in massage and invalid cookery is systematically provided, and all examinations are conducted by an outside examiner. The Guardians have now determined to erect an operating theatre with a view to the institution taking rank as a general hospital. The winner of the Frater Medal was Miss Lily Wignall.

A committee of the Guardians of the West Derby Union has formulated a scheme under which nurses and resident officers will not be called on to work more than fifty-six hours a week. It is much to be hoped that the Guardians will approve the scheme, which is, we believe, the first attempt to tackle the burning question of nurses' hours in Poor-Law institutions. It is an exceedingly moderate proposal, and deserves to be carried.

It is to Wales that maternity workers should go to study ideal conditions. Miss Rowlands, of the North Wales Nursing Association, has been describing the village of Penrhynside, on a hill near Llandudno, where the babies are all breast-fed, and sleep in their own cots day and night. The seventy-one districts affiliated to the association provide an efficient midwifery service combined with general nursing, and the ante-natal side of the work receives special attention. It is found that 60 per cent. of the children of the present day have defective teeth, babies before they are two often suffer from dental caries, and this is believed to be due to bad feeding of the mother before the child is born as well as improper feeding of the child before twelve months old. In North Wales the nurses are doing admirable preventive work in this connection.

An Advisory Committee has been appointed by the Ministry of Reconstruction to deal with the provision of mothers' helps for women unable on account of sickness and in child-birth to attend to their children and domestic duties. The Medical Officer of Health for St. Pancras is serving on the committee, the need being particularly urgent in this crowded centre.

The Association for Promoting the Training and Supply of Midwives is making an appeal for help in the valuable work it is doing in assisting suitable applicants to take training as midwives. Since the period of training was lengthened the cost is now close on £50, and though some candidates can subsequently refund part of the money granted, there are many taking up work among the poor who are unable to do so. It is anticipated there will be a great demand for midwifery training among demobilised workers, and the need for more midwives is among the pressing problems of the day. No suitable applicant ought to be deterred by lack of means from adopting this career. Cheques may be sent to the Hon. Treasurer at the offices of the Association, Dacre House, Dean Farrar Street, Westminster, S.W. 1, where also applications for assisted training can be entertained.

A small pamphlet has been issued by the National Food Reform Association (price 1s. 3d.), entitled "Dietaries." It is intended for secondary schools, colleges, hostels, clubs, etc. It contains a few recipes by Miss Dorothy Moore, L.C.A., and some remarks by Charles E. Hecht, M.A., M.C.A., on dietetic subjects, with a little matter reprinted from other sources. The cost of two dietaries for children is worked out at war prices, and each averages about 8s. per head per week. The little book is not too well arranged, and cannot be considered likely to add to the reputation of the association responsible for its production.

